# Reversible Nephrogenic Diabetes Insipidus Induced by Lithium: A Case Report

**DOI:** 10.1155/crin/9998067

**Published:** 2026-04-03

**Authors:** Sevil Uygun İlikhan, Gülin Dilken, Gökhan Hazıroğlu, Sinem Ülke, Selma Karaahmetoğlu

**Affiliations:** ^1^ Department of Internal Medicine, Ankara Bilkent City Hospital, Ankara, Turkey

**Keywords:** hypernatremia, lithium, nephrogenic diabetes insipidus, renal function tests, thiazides

## Abstract

Lithium is an effective mood stabilizer but may cause nephrogenic diabetes insipidus (NDI) by impairing the renal collecting duct response to arginine vasopressin (AVP). We report a 52‐year‐old woman on long‐term lithium therapy who presented with diarrhea, fatigue, polyuria, and confusion. Initial evaluation showed hypernatremia (serum sodium 156–159 mmol/L), low urine osmolality (101 mOsm/kg) despite serum osmolality of 286 mOsm/kg, daily urine output of 6.5–7.5 L, and a lithium level of 1.65 mmol/L. Renal function was preserved. Intravenous 5% dextrose was administered for free‐water replacement. Bicarbonate and potassium supplementation were initiated based on blood gas and biochemical findings consistent with metabolic acidosis (pH: 7.33 and serum bicarbonate: 22.1 mmol/L) and hypokalemia, requiring potassium supplementation. Lithium was discontinued, and a thiazide‐containing regimen was initiated. Without desmopressin, serum sodium normalized to 140 mmol/L within 72 h, urine output decreased to approximately 2 L/day, and mental status fully recovered. This case demonstrates that timely recognition and management of lithium‐induced NDI may allow recovery of urinary concentrating ability.

## 1. Introduction

Lithium is widely regarded as one of the most effective mood stabilizers for the treatment of bipolar and schizoaffective disorders. Despite its well‐established efficacy, its long‐term use is limited by a narrow therapeutic index and several dose‐related adverse effects, particularly on renal function. Chronic lithium exposure reduces the responsiveness of the renal collecting ducts to arginine vasopressin (AVP) and is associated with impaired urinary concentrating ability and polyuria [1, 2]. As a result, patients may develop nephrogenic diabetes insipidus (NDI), characterized by excessive excretion of dilute urine.

Earlier pathophysiological models emphasized inhibition of adenylyl cyclase and cyclic adenosine monophosphate signaling as the primary mechanism underlying lithium‐induced NDI. However, accumulating evidence suggests that lithium‐induced NDI may also develop through alternative pathways, including impaired aquaporin‐2 trafficking, epithelial sodium transport dysfunction, and direct tubular cell effects, independent of adenylyl cyclase activity [1, 2]. Clinically significant NDI has been reported in approximately 10%–15% of patients receiving long‐term lithium therapy [3]. Here, we present a case of reversible lithium‐induced NDI with rapid clinical improvement following discontinuation of lithium and supportive treatment.

## 2. Case Presentation

A 52‐year‐old woman with paranoid schizophrenia had been receiving long‐term maintenance therapy with clozapine, quetiapine, aripiprazole maintena, and lithium carbonate (900 mg/day). She presented to the emergency department with a 10‐day history of diarrhea, fatigue, excessive urination, and confusion.

On admission, the patient was confused, partially cooperative, and moderately dehydrated. Vital signs revealed a blood pressure of 108/51 mmHg and a heart rate of 100 beats per minute. The oral mucosa was markedly dry. Laboratory evaluation demonstrated severe hypernatremia, with serum sodium levels ranging from 156 to 159 mmol/L. Urine osmolality was inappropriately low at 101 mOsm/kg despite a serum osmolality of 286 mOsm/kg. Daily urine output ranged between 6.5 and 7.5 L. Serum lithium concentration was elevated at 1.65 mmol/L. Renal function was preserved (serum creatinine 0.98 mg/dL and estimated glomerular filtration rate 67 mL/min/1.73 m^2^).

The patient received intravenous 5% dextrose for free‐water replacement. Bicarbonate and potassium supplementation were initiated based on blood gas and biochemical findings consistent with metabolic acidosis (pH: 7.33 and serum bicarbonate: 22.1 mmol/L) and hypokalemia, requiring potassium supplementation. Lithium therapy was discontinued. To reduce polyuria, a fixed‐dose combination tablet containing ramipril 2.5 mg and hydrochlorothiazide 12.5 mg (Delix Plus 2.5/12.5 mg) was initiated, with the therapeutic intent focused on the thiazide component.

Within 72 h, serum sodium normalized to 140 mmol/L without the use of desmopressin, urine output decreased to approximately 2 L/day, and mental status returned to baseline. The clinical course following lithium discontinuation, including rapid normalization of serum sodium levels and marked reduction in urine output, is summarized in Figure 1. Written informed consent was obtained from the patient for publication of this case report and accompanying images.

FIGURE 1Clinical course following lithium discontinuation. (a) Serum sodium levels demonstrating rapid normalization within 72 h without desmopressin. An axis break is used to improve visualization of changes in the lower range. (b) Daily urine output showing marked reduction after the initiation of supportive therapy and thiazide treatment.

**Figure:**
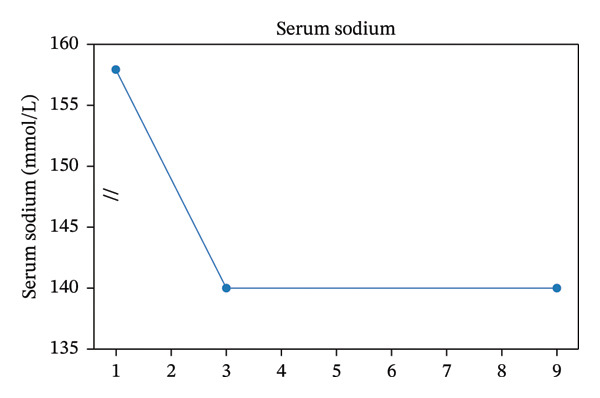
(a)

**Figure:**
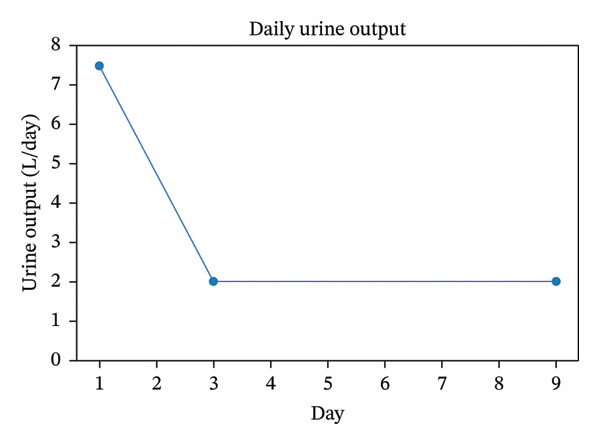
(b)

## 3. Discussion

NDI is the most common renal adverse effect associated with long‐term lithium therapy. The condition results from impaired renal responsiveness to AVP, leading to the excretion of large volumes of dilute urine [1]. Although earlier studies emphasized inhibition of adenylyl cyclase and cyclic AMP signaling, lithium‐induced NDI is now understood to involve multiple mechanisms, including aquaporin‐2 dysregulation, epithelial sodium transport abnormalities, and direct tubular cell toxicity [2, 4].

In the present case, severe hypernatremia accompanied by inappropriately dilute urine and marked polyuria strongly supported the diagnosis of lithium‐induced NDI. Thiazide diuretics reduce urine output in NDI by inducing mild extracellular volume contraction, which enhances proximal tubular sodium and water reabsorption and decreases distal tubular fluid delivery [5]. This mechanism explains the rapid reduction in urine output observed in our patient following the initiation of thiazide therapy.

Amiloride is frequently recommended in lithium‐induced NDI because it limits lithium entry into principal cells via epithelial sodium channels [6]. However, amiloride was not initiated in this patient because rapid clinical improvement was achieved after discontinuation of lithium and thiazide–based therapy, and concerns existed regarding potential electrolyte disturbances during acute correction.

The acute development of lithium toxicity in a patient previously on maintenance therapy may be explained by intercurrent gastrointestinal illness with diarrhea and dehydration, which can reduce renal lithium clearance and promote lithium accumulation [3, 7]. In addition, reduced free‐water intake during this period likely contributed to the development of hypernatremia in the setting of polyuria [5].

It should be noted that lithium‐induced NDI does not invariably present with hypernatremia, particularly in patients with adequate access to free water. Early recognition and discontinuation of lithium may allow partial or complete recovery of urinary concentrating ability, especially when intervention occurs before chronic structural renal damage develops [7, 8]. Routine biochemical monitoring and close collaboration between psychiatry and nephrology teams remain essential for the safe long‐term use of lithium.

## 4. Conclusion

Polyuria and electrolyte disturbances in patients receiving lithium therapy should raise suspicion for NDI. Prompt recognition and discontinuation of lithium, together with supportive management, may result in recovery of urinary concentrating function. This case highlights the importance of regular monitoring and interdisciplinary care to minimize renal complications associated with lithium therapy.

## Funding

No funding was received for this manuscript.

## Ethics Statement

Ethical approval was not required for this single‐patient case report according to institutional policy. Written informed consent was obtained from the patient for publication of the case details and accompanying images.

## Conflicts of Interest

The authors declare no conflicts of interest.

## Data Availability

The data that support the findings of this study are available upon request from the corresponding author. The data are not publicly available due to privacy or ethical restrictions.

## References

[1] Bedford J. J. , Weggery S. , Ellis G. et al., Lithium-Induced Nephrogenic Diabetes Insipidus: Renal Effects of Amiloride, Clinical Journal of the American Society of Nephrology. (2008) 3, no. 5, 1324–1331, 10.2215/cjn.01640408, 2-s2.0-53749093880. 18596116 PMC2518801

[2] Grünfeld J. P. and Rossier B. C. , Lithium Nephrotoxicity Revisited, Nature Reviews Nephrology. (2009) 5, no. 5, 270–276, 10.1038/nrneph.2009.43, 2-s2.0-67650360203. 19384328

[3] Davis J. , Desmond M. , Berk M. et al., Lithium and Nephrotoxicity: a Literature Review of Approaches to Clinical Management and Risk Stratification, BMC Nephrology. (2018) 19, no. 1, 10.1186/s12882-018-1101-4, 2-s2.0-85056119478. PMC6215627 30390660

[4] Bockenhauer D. and Bichet D. G. , Pathophysiology, Diagnosis and Management of Nephrogenic Diabetes Insipidus, Nature Reviews Nephrology. (2015) 11, no. 10, 576–588, 10.1038/nrneph.2015.89, 2-s2.0-84941994501. 26077742

[5] Sands J. M. and Bichet D. G. , Nephrogenic Diabetes Insipidus, Clinical Journal of the American Society of Nephrology. (2006) 1, no. 3, 539–548, 10.2215/CJN.04201205.17699257

[6] Eustatia-Rutten C. F. , Tamsma J. T. , and Meinders A. E. , Lithium-Induced Nephrogenic Diabetes Insipidus, The Netherlands Journal of Medicine. (2001) 58, no. 3, 137–142, 10.1016/s0300-2977(00)00104-2, 2-s2.0-0035120108. 11246113

[7] Aiff H. , Attman P. O. , Aurell M. et al., Renal Side Effects of Lithium: a 20-year follow-up Study, Journal of Psychopharmacology. (2019) 33, no. 9, 1150–1156.

[8] Markowitz G. S. and Radhakrishnan J. , Lithium Nephrotoxicity: an Update on Pathophysiology and Management, Kidney International. (2020) 97, no. 6, 1086–1098.

